# Insights from natural history collections: analysing the New Zealand macroalgal flora using herbarium data

**DOI:** 10.3897/phytokeys.30.5889

**Published:** 2013-11-26

**Authors:** Wendy A. Nelson, Jennifer Dalen, Kate F. Neill

**Affiliations:** 1 National Institute of Water and Atmospheric Research, Private Bag 14-901, Wellington 6241, New Zealand; 2 School of Biological Sciences, University of Auckland, Private Bag 92-019, Auckland 1142, New Zealand; 3 Museum of New Zealand Te Papa Tongarewa, P.O. Box 467, Wellington 6011, New Zealand

**Keywords:** Herbarium, macroalgae, Museum of New Zealand Te Papa Tongarewa, regional floras

## Abstract

Herbaria and natural history collections (NHC) are critical to the practice of taxonomy and have potential to serve as sources of data for biodiversity and conservation. They are the repositories of vital reference specimens, enabling species to be studied and their distribution in space and time to be documented and analysed, as well as enabling the development of hypotheses about species relationships. The herbarium of the Museum of New Zealand Te Papa Tongarewa (WELT) contains scientifically and historically significant marine macroalgal collections, including type specimens, primarily of New Zealand species, as well as valuable exsiccatae from New Zealand and Australia. The herbarium was initiated in 1865 with the establishment of the Colonial Museum and is the only herbarium in New Zealand where there has been consistent expert taxonomic attention to the macroalgae over the past 50 years. We examined 19,422 records of marine macroalgae from around New Zealand collected over the past 164 years housed in WELT, assessing the records in terms of their spatial and temporal coverage as well as their uniqueness and abundance. The data provided an opportunity to review the state of knowledge of the New Zealand macroalgal flora reflected in the collections at WELT, to examine how knowledge of the macroalgal flora has been built over time in terms of the number of collections and the number of species recognised, and identify where there are gaps in the current collections as far as numbers of specimens per taxon, as well as with respect to geographical and seasonal coverage.

## Introduction

Herbaria and natural history collections (NHC) are critical to the practice of taxonomy. They are the repositories of vital reference specimens, enabling species to be studied and their distribution in space and time to be documented and analysed, as well as enabling the development of hypotheses about species relationships. Krishtalka and Humphrey (2000) describe natural history museums as “sentinel observatories of life on Earth” and also “stewards of its future”. Repeatable and testable biological sciences are reliant on taxonomy and vouchered specimens. Within the past decade or so, there has been an increasing recognition of the value of collections in the analysis of biodiversity, with interest in their potential applications for example in conservation and ecology, inferring threats associated with anthropogenic change (e.g. McCarthy 1998, Shaffer et al. 1998, Ponder et al. 2001, Graham et al. 2004, Frey 2009, Newbold 2010, Pyke and Ehrlich 2010, Johnson et al. 2011, Tomizuka et al. 2012, Ward 2012). A number of studies have explored the ways in which NHC may be used to evaluate responses of biota to climate change, including examination of apparent shifts in species ranges, detecting the presence of possible introduced species, and prediction of the future changes in species distributions and patterns of species richness under future climate scenarios (e.g. Graham et al. 2004, Johnson et al. 2011).

There are many challenges when using NHC for analyses of biota, particularly the potential sources of errors, accuracy and biases (Graham et al. 2004, Boakes et al. 2010, Newbold 2010, Pyke and Ehrlich 2010, Huisman and Millar 2013). On the one hand, NHC provide an “unambiguous record of a taxon at a particular place and time” with the advantage that vouchered material enables identifications to be verified and additionally taxonomic concepts can be updated (Johnson et al. 2011). However there are significant issues about the quality of the identifications, that is, whether these have been provided by a subject specialist, and also whether taxonomic concepts, changes in nomenclature, and synonymies are being updated.

In terms of spatial and temporal data, older collections tend to be geo-referenced post-collection which may introduce location errors. The data associated with specimens are often highly variable as far as the level of detail provided, for example, the precision of the locality of the collection, habitat information, associated species, collection method, and whether multiple collections were made from within different habitats within a site. Some early collections (19^th^ century, early 20^th^ century) have only the year or month of collection provided with the specimen. Biases affect different aspects of the collections. Spatial biases can result from the position of access roads and settlements, particularly in the case of coastal collecting. Access to collection methods and equipment is also critical, for example, in the case of marine macroalgae in New Zealand there are large sections of coastline where access is only possible from the sea, and thus the use of boats is critical. Weather can have a significant impact on accessibility of sites and can lead to seasonal biases. For an important part of the flora sampling is only possible via SCUBA and via dredge equipment for deep-water samples. The location of active collectors has an impact on the number of collections obtained from particular regions, as does the perception of areas being of specific interest. Temporal biases can result from experts working actively on particular taxonomic groups, as well as from particular curatorial practices and personal interests (e.g. discarding damaged individuals, only accessioning a certain number of individuals) (Ward 2012).

Collections result from targeted investigations as well as from opportunistic sampling. Sometimes remote areas are infrequently visited but have detailed and thorough collections because major effort is required to reach the area and very deliberate collections are undertaken. There are inevitably biases as far as which species are collected, with the potential for larger or more conspicuous species to be over-represented and with smaller or more difficult to collect species under-represented. In addition, sometimes common species are under-collected whereas rare or unusual species are collected more frequently. Graham et al. (2004) consider that “nonrepresentative sampling in environmental space remains the most difficult source of error to detect and correct”. The material in NHCs only provide presence data, establishing that the species was present at that locality when collected. Interpretation of species absences is complex - the species may not have been at the locality, or was not collected, or not detected.

## Macroalgal collections from New Zealand

In the mid to late 19th century there was a period of research on New Zealand algae by overseas research workers, particularly W.H. Harvey (Trinity College, Dublin), J.D. Hooker (Kew, London) and J.G. Agardh (Botanical Museum, Lund). In some cases material was deposited in New Zealand collections, e.g. collections made by William Colenso were sent to Hooker and Harvey and some material retained in New Zealand; collections made by Sven Berggren in 1874 were sent to Agardh with some duplicate material returned to New Zealand (Bagnall 1970). However this was followed by many years when there was very little activity on the description or study of New Zealand algae. Collections made by New Zealanders rather than foreign visitors began with the work of R.M. Laing and W.A. Scarfe, and compilations of species were prepared by Laing (e.g. Laing 1900, 1902, 1909, 1926, 1930).

From the mid-1930s very significant collections were made by Victor Lindauer (Cassie 1971, Cassie Cooper 1995) who corresponded with several international phycologists and also received specimens from Eileen Willa on Stewart Island. From the 1930s onwards Lucy Cranwell and Lucy Moore, at that time students at Auckland University College, made important collections and ecological observations. The establishment of algal research within Botany Division of the Department of Scientific and Industrial Research (DSIR) saw the development of collections by Moore and Nancy Adams. In the late 1940s both Dr T. Levring and Professor G.F. Papenfuss visited New Zealand. The collections made by Papenfuss provided material for many of his graduate students who made major contributions to the understanding of the New Zealand macroalgal flora (e.g. Wagner 1954, Norris 1957, Sparling 1957, Hommersand 1963). Taxonomic treatments of the flora began to be published (e.g. Levring 1955, Chapman 1956, 1969, 1979, Lindauer et al. 1961, Chapman and Dromgoole 1970, Chapman and Parkinson 1974), but to date there has been no comprehensive treatment of the macroalgal flora.

The major macroalgal collections are held in New Zealand in the herbaria of the Museum of New Zealand Te Papa Tongarewa (WELT), Landcare Research Manaaki Whenua (CHR) and at the Auckland Museum (AK) (Thiers 2012). However, WELT is the only herbarium in New Zealand where there has been consistent expert taxonomic attention to the macroalgae over the past 50 years (Nancy Adams 1969–1987, Wendy Nelson 1987–2002, Jenn Dalen 2002-present). The herbarium was initiated in 1865 with the establishment of the Colonial Museum (1865–1906), subsequently known as the Dominion Museum (1906–1973), National Museum of New Zealand (1973–1992) and the Museum of New Zealand Te Papa Tongarewa (Te Papa) (from 1992). Algal specimens were received into the collections in the 1860s and 1870s, from the inception of the herbarium, including a collection from the British Museum of more than 200 algal specimens from throughout the British Isles dating from 1806-1860 and algal specimens from the Thompson/J.G. Baker herbarium (Nelson et al. 1998). The Te Papa herbarium contains scientifically and historically significant marine macroalgal collections including, type specimens, primarily of New Zealand species, as well as valuable exsiccatae from New Zealand and Australia (Nelson et al. 1998).

In the absence of a complete flora, there has been considerable recent effort directed to compiling and updating lists of currently accepted names and the taxonomic hierarchy, with published lists produced as part of the Species 2000 project documenting the New Zealand biota (Broady et al. 2012, Harper et al. 2012, Nelson 2012), and also updated current lists provided on the Te Papa website (e.g. Dalen and Nelson 2013a-c). Much of New Zealand macroalgal taxonomic and biogeographic literature is based on the WELT collections including Adams (1994) and a series of regional floral lists (Adams 1972, Adams et al. 1974, South and Adams 1976, Nelson and Adams 1984, Adams and Nelson 1985, Hay et al. 1985, Nelson and Adams 1987, Nelson et al. 1991, Nelson et al. 1992, Neale and Nelson 1998, Nelson et al. 2002) based on targeted collections. In addition some specific projects were undertaken to improve collections and knowledge of the flora (e.g. coralline algae, Harvey et al. 2005, Broom et al. 2008, Farr et al. 2009; macroalgae from soft sediment environments, Neill et al. 2012; Ulvaceae, Heesch et al. 2007, 2009).

The WELT collections have been databased over a period of ca. 15 years. The recent focus within the herbarium has been on improving collection data and checking the dataset for errors, particularly grooming collection date data and mapping and verifying locality data. The collections at both AK and CHR have not been fully databased to date and have not received the level of scrutiny and identification that has been directed to the WELT collections. The AK and CHR collections currently do not have specialist marine phycologists associated with the collections. Because WELT collections have received expert identification and curation, they have been used as the primary source of data on the distributions of marine macroalgae for a number of research projects and government databases (e.g. Booth et al. 2006) and WELT is also where voucher specimens have been deposited (e.g. for the Marine Invasives Taxonomic Service, contracted to NIWA by the Ministry for Primary Industries).

## The purpose of this study

The grooming and updating of the database has provided an opportunity to review the state of knowledge of the New Zealand macroalgal flora reflected in the collections at WELT, and to ask a series of questions. Which regions of New Zealand are represented by the most comprehensive collections/least comprehensive collections? How has knowledge of the macroalgal flora built over time in terms of the number of collections and the number of species recognised? Are there patterns that can be discerned in the collection history and coverage? What proportion of the flora is represented by sufficient individuals for study and comparative investigation (number of specimens, geographic range, seasonal distribution)? Do these collections have the potential to enable other types of biodiversity analyses?

## Materials and methods

The data presented in this paper are drawn from the database of the Te Papa herbarium. Definitions for terms used in this paper are provided in Table 1. Data were assembled following several steps:

**Table 1.** Definitions of terms used in this paper.

Term	Definition
Collecting event	For each region collections were sorted by year, then collection date and precise location. Each unique combination of date and precise location was treated as a collection event.
Duplicate records	Specimen duplicates, i.e. same taxon with identical collection data. (Only 1 example of duplicate sheets (e.g. labelled a–c) was retained; duplicates with different registration numbers were removed.)
Season	Collection dates were grouped by month and allocated to seasons as follows:<br/> December-February = summer; March-May = autumn; June-August = winter; September-November = spring
Record	Single packet, box or specimen sheet
Taxon	Name used in database which includes identifications to family, genus and species level as well as tag names (informal names assigned usually in preliminary stages of investigations or for entities recognised in the field)

### History of data and specimen information verification

Until the early-1990s, herbarium specimen data were available from the specimen labels and a hard copy register. The first electronic database system at Te Papa, Te Kahui, was custom-designed and implemented in 1993. Data were retrieved from the specimen labels and entered into the system by trained data-entry technicians. Where appropriate, extra information was sourced - the majority of this being latitude and longitude information derived from maps (NZMS 260 series). Most of the existing New Zealand algal specimens in the collection had an electronic record completed by ca. 2001. However, the record error rate was relatively high – in the order of 30-40%, with respect to coordinate data and locality information.

In 2005, all of the museum’s electronic records were migrated to an electronic collection information management system, KE EMu® (referred to as EMu), a relational database customised for museum collections. With the implementation of the new database, there was scope to improve the quality of information recorded. Features of EMu, such as sophisticated search functions and global updating options, have facilitated cross-checking for consistency in the locality records, use of place names and collector details. The verification and grooming of the algal data (e.g. cross checking of longitude and latitude, consistency of locality records, use of place names, collector details, identifying missing data fields and locating information where available) has become a core collection management activity since this time. However, much of this grooming effort has been somewhat opportunistic (for example, new acquisitions prompt a cross-checking of data for similar/nearby locations). Several special projects and requests for data have prompted more comprehensive data verification efforts. As part of this, several thousand backlog algal specimens were identified and databased; the database component of the work further prompted refinement to the consistency and accuracy of the locality data. Attention to the application of taxonomic concepts and names across the collection was also undertaken as part of this work.

### Taxonomic framework

In the absence of a published flora, a current species names list and taxonomic hierarchy is maintained on the Te Papa website (http://www.tepapa.govt.nz/ - Dalen and Nelson 2013a–c). Changes to current taxonomic names and classification have been drawn from primary literature and updated into Te Papa’s database and the application of name changes to the collections has also been part of this effort.

### Regional categorisation

**Figure 1.**
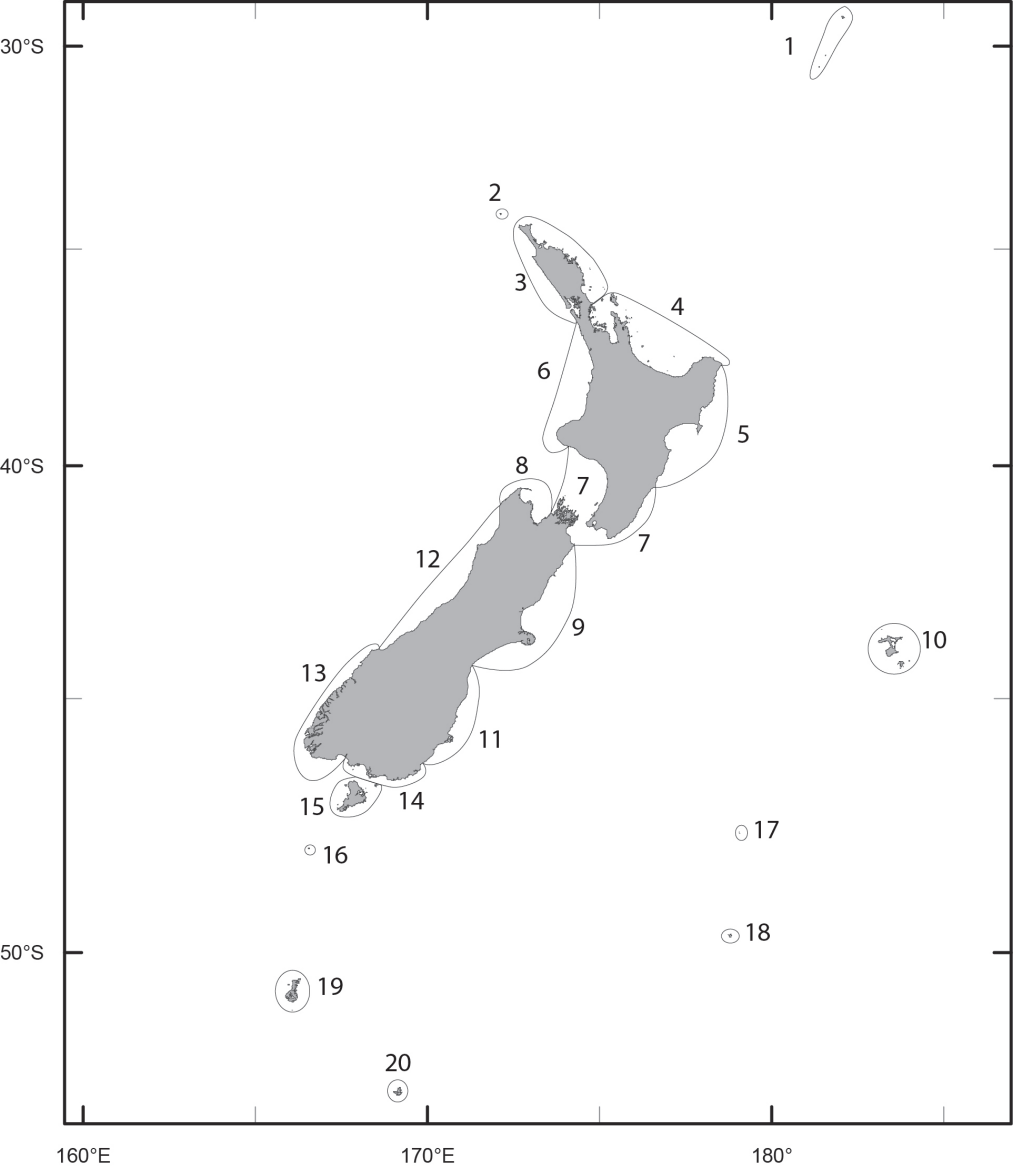
Map of New Zealand indicating the boundaries of the regions investigated in this study (**1** Kermadec Is **2** Three Kings Is **3** North I. (NI) North **4** NI Bay of Plenty (BOP) **5** NI East **6** NI West **7** Wairarapa-Cook **8** South I. (SI) Northwest (NW) **9** SI Kaikoura **10** Chatham Is **11** SI Southeast (SE) **12** SI Westland **13** SI Fiordland **14** SI Southern **15** Stewart I. **16** Snares Is **17** Bounty Is **18** Antipodes Is **19** Auckland Is **20** Campbell I).

Figure 1 illustrates the regional boundaries applied in this exercise. The boundaries reflect a combination of biogeographic boundaries in previously published accounts of the marine biota (e.g. Adams 1994, Nelson 1994, Shears et al. 2008), as well as province definitions employed by Te Papa for the plant collections.

### Download of data from Te Papa database

All New Zealand marine algal records were searched and grouped by latitude and longitude coordinates corresponding to the regions as defined above (20 categories) in Te Papa’s database. Records, current to December 2011, were exported by region into Excel spreadsheets. Table 2 summarises the data used in the analyses undertaken. The number of taxa includes all recognised entities present in the collections, including some that have been recognised as distinct at a family, genus or species level but are currently unnamed. It is important to note that the publicly accessible flora lists (Dalen and Nelson 2013a-c) only include published names, including some published tag names, that not all published taxa are represented in the WELT collections, and that there are more taxa recognised as being distinct than have been published currently. The Green algae or Division Chlorophyta includes data for three classes, Prasinophyceae, Ulvophyceae, and Trebouxiophyceae. (There are no marine macroalgal Chlorophyceae represented in Te Papa’s collections.) The Brown algae or Ochrophyta include members of the classes Chrysomerophyceae, Xanthophyceae, and Phaeophyceae, and the Red algae or Rhodophyta are represented by members of 4 classes, Compsopogonophyceae, Stylonematophyceae, Bangiophyceae, and Florideophyceae.

**Table 2.** Summary of the specimen records and taxa analysed in dataset:

	Greens	Browns	Reds	Total
Number of records	2,859	5,495	17,043	25,397
Number of unique records	2,213	4,580	12,629	19,422
Number of taxa	118	199	679	996
Number of classes	3	3	4	10
Number of orders	7	13	20	40
Number of families	16	30	52	98
Number of genera	**25**	**75**	210	310

## Results and interpretation

**Figure 2.**
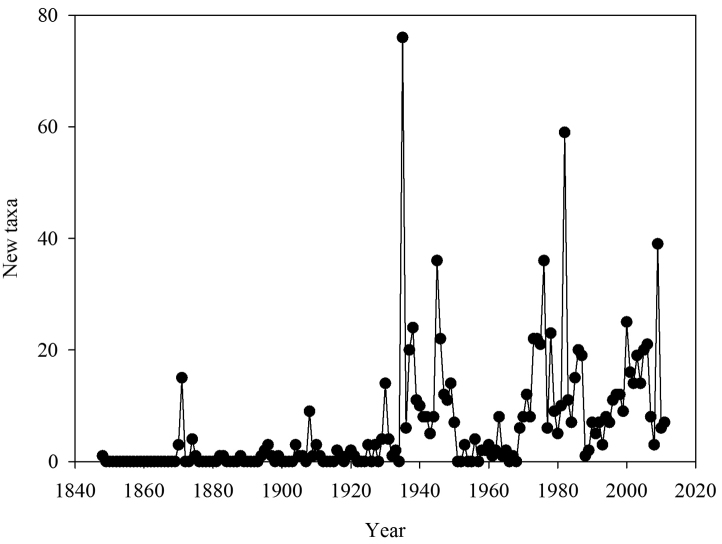
Number of new taxa represented in the collection by year of collection.

**Figure 3.**
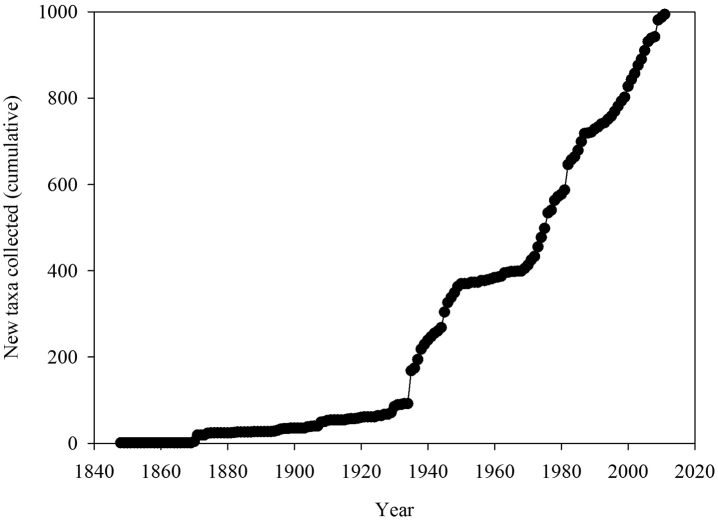
Cumulative total of taxa in the collections.

**Figure 4.**
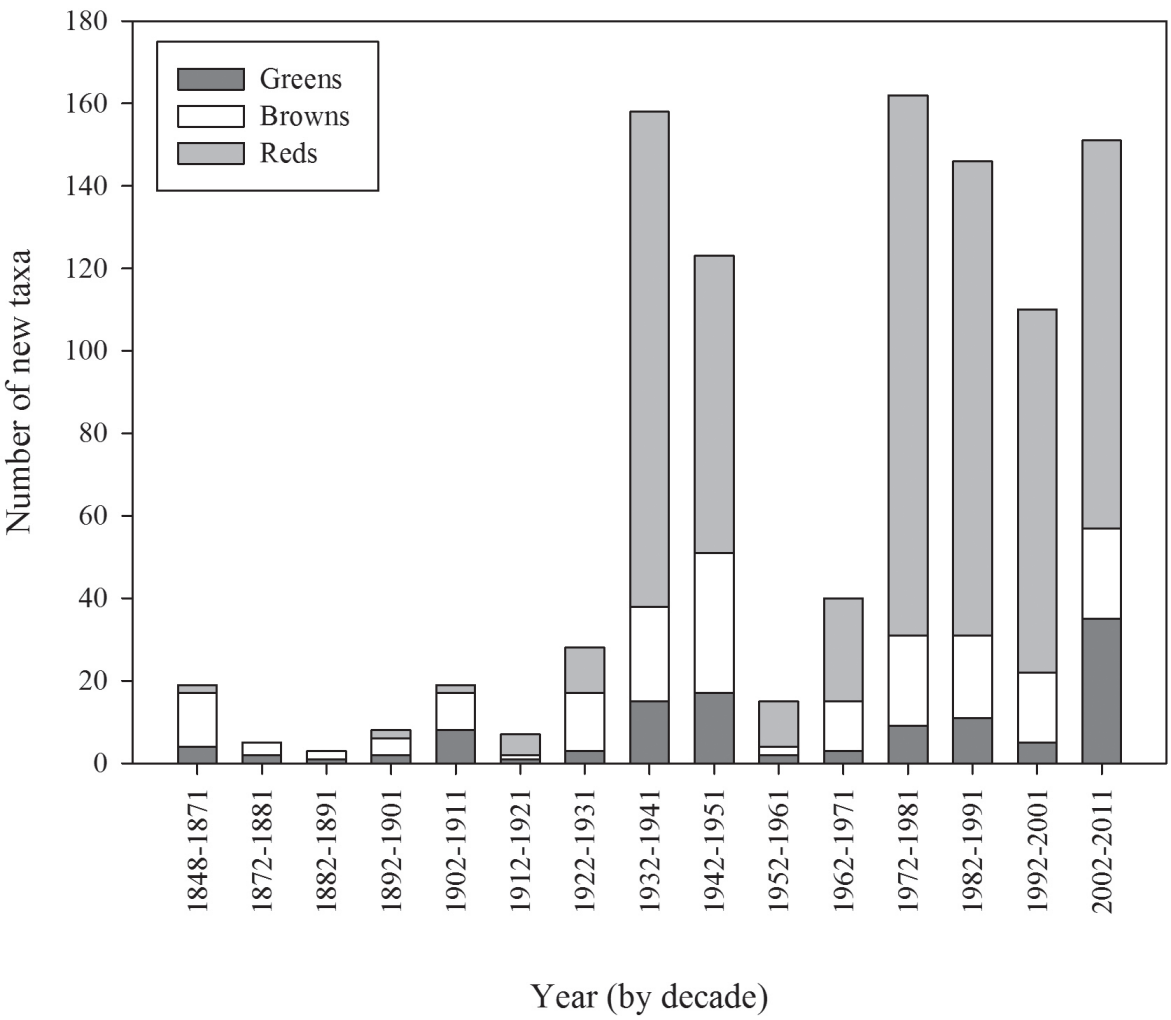
Number of taxa in the collections by decade of collection and division (green, brown, and red algae).

**Figure 5.**
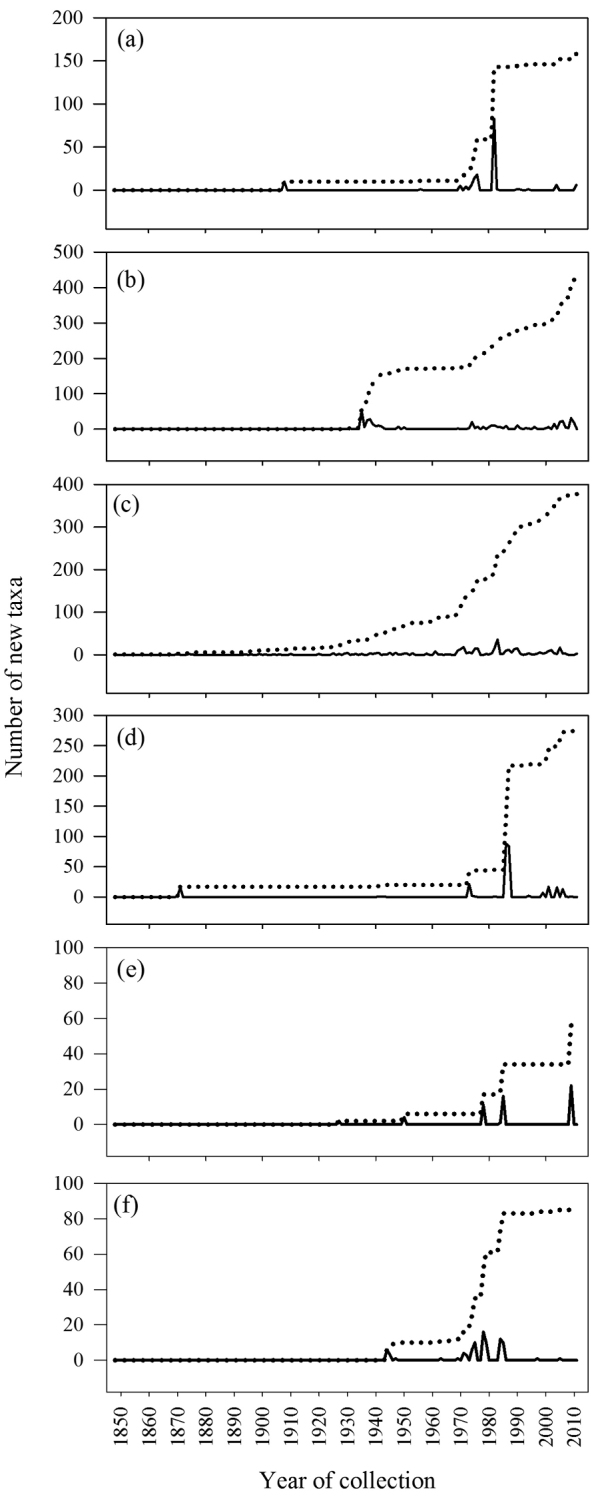
Annual (solid line) and cumulative (dashed line) new taxa from selected regions: **a** Kermadec Is **b** NI North **c** Wairarapa-Cook **d** Chatham Is **e** Bounty Is **f** Campbell I.

The number of new taxa represented in the collection by year of collection is presented in Figure 2, the cumulative total of taxa in the collections in Figure 3, and the number of taxa in the collections by decade of collection broken into divisions (red, brown, and green algae) in Figure 4. The surge in the number of collections around the 1870s reflects the material collected by both S. Berggren and H.H. Travers that was sent to Lund for examination by J.G. Agardh with duplicate material returned to New Zealand. Most specimens lodged prior to the 1930s were collected by W.A. Scarfe and R.M. Laing. In 1935 Josephine Tilden from the University of Minnesota, and a group of associates, visited New Zealand collecting in the Bay of Islands as well as on Stewart Island, and material was distributed as “South Pacific Plants”. V.W. Lindauer, the school teacher at Russell, Bay of Islands, was introduced to seaweeds by Tilden, resulting in his major contributions to New Zealand phycology (Cassie 1971, Cassie Cooper 1995, Nelson and Phillips 1996). Lindauer assembled the Algae Nova-Zelandicae Exsiccatae (ANZE), consisting of 350 sheets and distributed in 14 fascicles between 1939 and 1953, incorporating his own collections, those of family and pupils and also material he received from Mrs Eileen Willa on Stewart Island. The peaks in the annual number of new taxa between 1930 and ca. 1955 are largely based on the Lindauer collections and exsiccatae. The peaks in collections and the upward surge in cumulative number of taxa from 1969 coincides with the arrival of Nancy Adams at WELT, with collecting primarily in the Wellington and Wairarapa regions. The increased availability and use of SCUBA resulted in new collections deposited by other marine research workers. During the 1980s and 1990s collections for the series of regional flora lists resulted in many new collections as well as new taxa. During the 2000s specific projects on elements of the flora (e.g. Bangiales (Nelson et al. 2001, 2003, 2005, 2006), Ulvaceae (Heesch et al. 2007, 2009), non-geniculate coralline algae (Harvey et al. 2005, Farr et al. 2009), macroalgae associated with soft sediments (Neill et al. 2012)) contributed to peaks in particular groups of algae. Figures 5a–f present the cumulative number of species recorded from selected regions (Kermadec Islands, NI North (Northern North Island), Wairarapa-Cook, Chatham Islands, Bounty Islands, Campbell Island), revealing the patterns of collecting history in greater detail.

Analysis revealed that many entities in the flora are known from very few records (Table 3). Of the 996 taxa in this analysis there are only 210 taxa in the collection for which there are more than 30 records (20 greens, 51 browns, 139 reds). Over the whole collection 17% of the taxon records are known from a single record and 44% from five or fewer records.

**Table 3.** Number of taxa and records by class of algae.

	Greens	Browns	Reds	Total
Number of taxa:	118	199	679	996
Number of taxa known from a single record:	26 (22%)	42 (21%)	97 (14%)	165 (17%)
Number of taxa known from 5 or fewer records	65 (55%)	84 (42%)	294 (43%)	443 (44%)
Number of taxa known from > 30 specimens	20 (17%)	51 (25%)	139 (20%)	210 (14%)
Number of taxa known from >100 records	3 (3%)	8 (4%)	15 (2%)	26 (3%)

**Figure 6.**
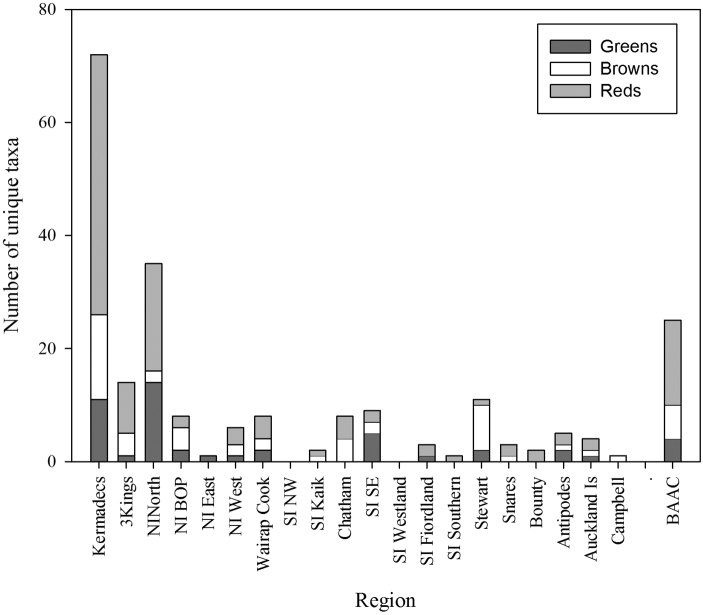
Number of taxa unique to each region by division (BAAC = Bounty, Antipodes, Auckland, Campbell Islands).

The data available for each of the 20 regions within New Zealand are summarised in Table 4. This lists the number of taxa, the composition of the flora in each region, the number of records that the data are based on, the composition of the flora by region in terms of the number of classes, orders and families represented, the number of years in which collections were made (of a potential 164 years between 1848-2011), and the seasons in which collecting events occurred. In some regions the collections have been made over an extended period, and span all seasons, whereas it is clear that some other regions are infrequently visited and no collections made in some seasons. Overall the impact of season is relatively modest with the number of collections from the winter months being only two thirds of the total from the summer months. Figure 6 summarises the number of taxa unique to each region by class.

**Table 4.** Distribution of macroalgal collections by region.

	Kermadec	3 Kings	NI North	NI BOP	NI East	NI West	Wairarapa_Cook	SI NW	SI Kaikoura	Chathams	SI SE	SI Westland	SI Fiordland	SI Southern	Stewart	Snares	Bounty	Antipodes	Auckland	Campbell
Region (Fig. 1)	1	2	3	4	5	6	7	8	9	10	11	12	13	14	15	16	17	18	19	20
Number of taxa	159	178	421	314	127	205	381	173	233	275	276	195	271	145	282	111	56	140	101	86
Number of greens	22	19	61	37	14	21	42	22	24	27	43	19	32	16	31	8	5	17	12	9
Number of browns	35	36	86	77	34	41	93	32	57	61	60	41	53	31	81	20	10	23	19	16
Number of reds	102	123	274	200	79	143	246	119	152	187	173	135	186	98	170	83	41	100	70	61
Regional taxa as % of total flora	16.1	18.0	42.5	31.7	12.8	20.7	38.5	17.5	23.5	27.8	27.9	19.7	27.4	14.6	28.5	11.2	5.7	14.1	10.2	8.7
Number of unique taxa	72	14	35	8	1	6	8	0	2	8	9	0	3	1	11	3	2	5	4	1
Number of records	532	714	2998	1372	339	645	3222	565	659	1527	1070	918	1588	407	1289	310	147	552	349	219
Number of taxa known from: a single record (%)	63 (40)	61 (34)	104 (25)	93 (30)	40 (31)	77 (38)	73 (19)	49 (28)	97 (42)	50 (18)	86 (31)	33 (17)	69 (25)	52 (36)	61 (22)	46 (41)	22 (39)	52 (37)	36 (36)	38 (44)
Number of taxa known from 5 or fewer records (%)	131 (82)	134 (75)	254 (60)	237 (75)	116 (91)	177 (86)	208 (55)	144 (83)	207 (89)	170 (62)	222 (80)	143 (73)	180 (66)	130 (90)	195 (69)	97 (87)	52 (93)	105 (75)	83 (82)	76 (88)
Number of taxa known from > 30 specimens	0	1	10	4	0	0	14	0	0	0	0	1	3	0	0	0	0	0	0	0
Collecting events	92	74	707	420	84	226	957	93	261	196	253	110	272	84	272	59	28	79	84	46
Number of years collected	18	13	60	54	23	48	87	22	47	23	56	26	30	28	47	14	6	9	19	16
Springs collected	7	5	37	30	11	19	53	5	24	4	28	15	10	8	26	6	2	4	4	7
Summers collected	1	9	40	29	13	22	54	14	19	16	31	17	21	15	31	13	0	2	15	8
Autumns collected	9	3	33	32	5	25	55	8	18	7	20	7	10	8	19	1	4	4	3	5
Winters collected	5	0	26	24	5	22	47	3	21	5	17	6	6	7	14	1	0	0	2	1
Number of classes	5	6	8	5	4	5	6	5	7	6	6	4	5	5	7	4	5	6	6	5
Number of orders	25	30	36	30	24	29	31	28	33	32	33	29	30	25	32	26	19	29	27	25
Number of families	50	55	83	73	44	57	75	53	64	72	67	59	62	49	69	44	29	46	48	42

## Discussion

These analyses have provided an opportunity to review the state of knowledge of the New Zealand macroalgal flora and to investigate how well the current collections at Te Papa represent the macroalgal flora of New Zealand. The cumulative total of taxa in WELT shows that the flora is not reaching asymptote, suggesting that more discoveries are likely with further investigations of the flora. It is clear that some regions of New Zealand have received greater attention (number of records, collecting events) (Table 4), and as a consequence the flora is better understood in these areas (in particular Wairarapa-Cook, NI North). The number of collections available by region is in large part a consequence of the presence of active research workers, research institutions or programmes, as well as the accessibility of the coastline. The northern and southern island groups - Kermadec and Three Kings Islands, and Snares, Bounty, Antipodes, Auckland, Campbell Islands - are all difficult to reach, subject to inclement weather and are relatively infrequently sampled, i.e. collections made in fewer than 20 years in the 164 years since the first New Zealand collections were lodged in WELT (Table 4).

The knowledge of the flora in different regions has been built up in quite different ways. Macroalgae were first collected from the Kermadec Islands by New Zealand based scientists in 1908, although material had been collected in 1854 and 1874 by expeditions and lodged in European herbaria (Nelson and Adams 1984). It was not until collections were made by a trained phycologist in 1982 that the flora was more thoroughly understood (Fig. 5a). There have been no subsequent targeted collections from the Kermadec Islands with only occasional opportunistic collections deposited in WELT (Fig. 5a). This collection history, coupled with the fact that 82% of the flora is currently known from 5 or fewer records (Table 4), strongly suggests that the macroalgae of this region are under-represented in the collection. The flora of the Kermadec Islands differs markedly from the rest of New Zealand, with strong affinities to the warm-water regions of the Pacific and Indian Oceans (Nelson and Dalen in press). Of the 152 taxa recorded in our data from the Kermadec Islands, almost half of these are represented in the New Zealand region only in these northern islands (Figure 6).

The collection history of the NI North (Fig. 5b) and Wairarapa-Cook (Fig. 5c) are interesting to compare. These are the most diverse and intensely sampled regions. The Bay of Islands is a key collecting area in the NI North and has seen bursts of collecting activities by Tilden and Lindauer in particular. The collecting history of the Wairarapa-Cook region reflects the presence of phycologists and collectors, with a steady growth in the knowledge and representation of the flora over an extended period. The first collections of macroalgae from the Chatham Islands that formed the basis of a published account were made in 1863-64 and then in 1871, with further collections made by a German research expedition in 1897. However it was not until the mid-1980s that thorough and detailed collections were made of the flora (Fig. 5d). In the intervening 80-90 years there were only scattered and infrequent collections made on the islands (summarised in Nelson et al. 1991). Based on the number of collections the Chatham Islands flora is now relatively well represented at Te Papa, although 18% of the flora is still known from only a single record. The collecting history of the southern Bounty Islands (Fig. 5e) (which are only 1.3 km^2^ in area and 700 km distant from the nearest landmass) and Campbell Island (Fig. 5f) (113 km^2^ in area, also 700 km distant from the nearest landmass) reflect problems of access. This is also shown in the seasonal breakdown of collecting years (Table 4).

In terms of regional diversity the northern North Island has the most taxa recorded, contains the highest proportion of the total flora, and has the greatest phylogenetic diversity (as represented by the highest numbers of classes, orders and families present). Although there are almost 3000 specimen records from the region, 25% of the taxa from the area are known from a single record, and 60% from 5 or fewer records. The offshore Three Kings Islands and the southern islands (Snares, Bounty, Antipodes, Auckland and Campbell Islands) are represented by the smallest number of collections resulting from few collecting events. The mainland areas that have received the least collecting effort (fewest collection events) are North Island East, South Island Northwest, and the South Island Southern.

The proportion of the flora that is represented by a very small number of records is salutary, with ca. 44% of the flora known from 5 or fewer records (Table 3). When the data are examined by region (Table 4) the differences in the coverage of collections can be assessed. For phenological studies and comparative investigations it is important to have a number of specimens to evaluate variation and attributes that may be influenced by maturity, seasonality, and/or reproductive status. There are 210 taxa in the collections, identified to species, for which there are more than 30 records: in our view, this number of records provides sufficient individuals for such comparative studies. In terms of the application of NHC collections for understanding the responses of the flora to human-induced environmental changes, Johnson et al. (2011) consider that collections with “large numbers of common taxa are the most useful as time series for determining species level responses” although they note that such collections “typically have been perceived as of low priority for acquisition or curatorial effort”.

Our analyses have enabled us to test the quality of the data associated with the specimens. The data grooming exercises prior to these analyses have minimised location errors (e.g. latitude and longitude, place names) but opportunity for minor transcription errors still remains. The main issues affecting data quality are the level and standard of identification, which are directly influenced by the current state of the systematic knowledge of the flora. There have been few monographic studies of macroalgal taxa in New Zealand, but recent research across a range of orders has revealed new taxa, and the need for significant taxonomic revisions. These studies have also concluded that understanding the diversity in the flora is still in a discovery phase (e.g. Broom et al. 2004, Heesch et al. 2009, D’Archino et al. 2011). Although Te Papa’s collection data are the best available at present within New Zealand, our analyses have revealed that the macroalgal flora is currently poorly represented in terms of numbers of records for many taxa, as well as in the geographic and seasonal spread of specimens.

Although it is questionable whether the Te Papa collections constitute a comprehensive or sufficient baseline with which to evaluate change in the environment or in the flora composition, these herbarium specimens are a very significant source of data both for current biodiversity assessment and planning and also for future applications in biodiversity analysis, conservation and ecology. There are areas within the collection that have been developed from specific research programmes (e.g. collections for the regional floral list series, Bangiales, coralline algae, macroalgae from soft sediment environments, Ulvaceae) where the specimens have been collected in a systematic and targeted way, and in some cases can be associated with other key environmental data. These collections provide a reference baseline if there are opportunities for the regions or habitats to be resampled in the future. The analyses performed here are repeatable if the collection continues to receive the same attention, i.e., expert identification and application of current names, precision of data entry with respect to consistency of place names, and coordinate data

This analysis has identified gaps in the macroalgal collections, both taxonomically and geographically, and also data that can inform future collection development. A number of recent papers reviewing the role of NHCs have stressed the function of museums and herbaria as “part of the essential infrastructure of science” (Johnson et al. 2011) and their value to conservation biologists and ecologists for studying species’ distributions and abundance (Newbold 2010). Institutions faced with the expense of care and maintenance of NHCs need to have strategically focused research and collection development policies which identify the opportunities for their collections to serve not only research on biosystematics, distribution and evolution of biotas, but also to have wider applications for environmental and conservation science. Whilst opportunistic collections can be valuable in providing material to complement existing material, and have often resulted from collectors seeking to maximise field opportunities and access to infrequently visited areas, there is a need to move to a more systematic approach to the sampling of diversity to provide higher quality data. Ward (2012) recommends that NHCs “must become drivers of biodiversity science” and suggested four key priorities for NHCs – mass databasing, analysis of holdings, identification of ecological datasets, and repositories of ecological projects. We have addressed the first two of these priority areas and have also identified datasets within the Te Papa macroalgal herbarium that have potential to serve as baselines for future research. At present the herbarium is not equipped to serve as a repository for ecological projects. This aspect of future-focused work is challenging and considerable care will be needed to develop data protocols to record information about sampling effort, population size and other ecological attributes.
